# Supplementary material for: Abscisic Acid Affects Phenolic Acid Content to Increase Tolerance to UV-B Stress in Rhododendron chrysanthum Pall
Source: Int J Mol Sci. 2024 Jan 19;25(2):1234. doi: 10.3390/ijms25021234 (PMC10816200; doi:10.3390/ijms25021234)
Supplement: Supplementary file 1 [file ijms-25-01234-s001.zip › ijms-2811187-supplementary.pdf]

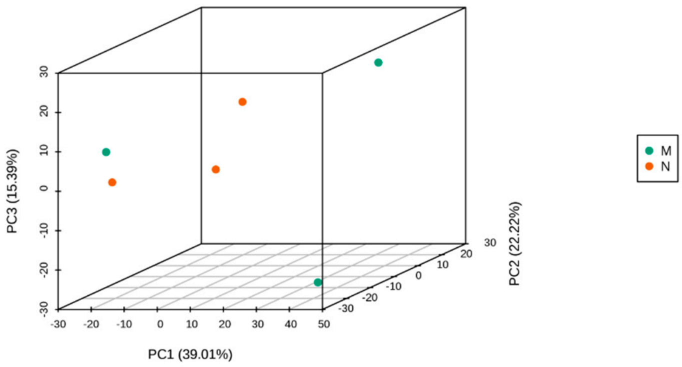
PC1 (39.01%)
PC2 (22.22%)
PC3 (15.39%)
M
N

(a)

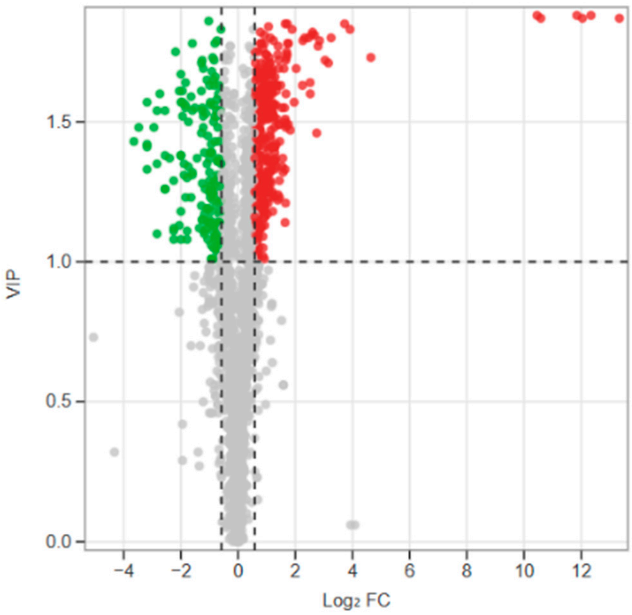
VIP
Log2 FC

(b)

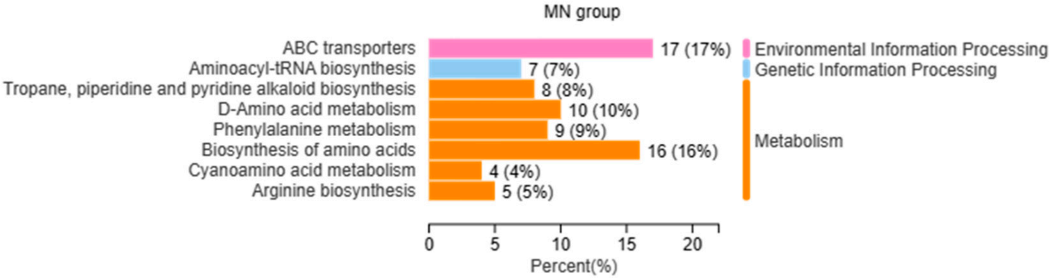
MN group
ABC transporters
Aminoacyl-tRNA biosynthesis
Tropane, piperidine and pyridine alkaloid biosynthesis
D-Amino acid metabolism
Phenylalanine metabolism
Biosynthesis of amino acids
Cyanoamino acid metabolism
Arginine biosynthesis
17 (17%)
7 (7%)
8 (8%)
10 (10%)
9 (9%)
16 (16%)
4 (4%)
5 (5%)
Environmental Information Processing
Genetic Information Processing
Metabolism
Percent(%)

(c)

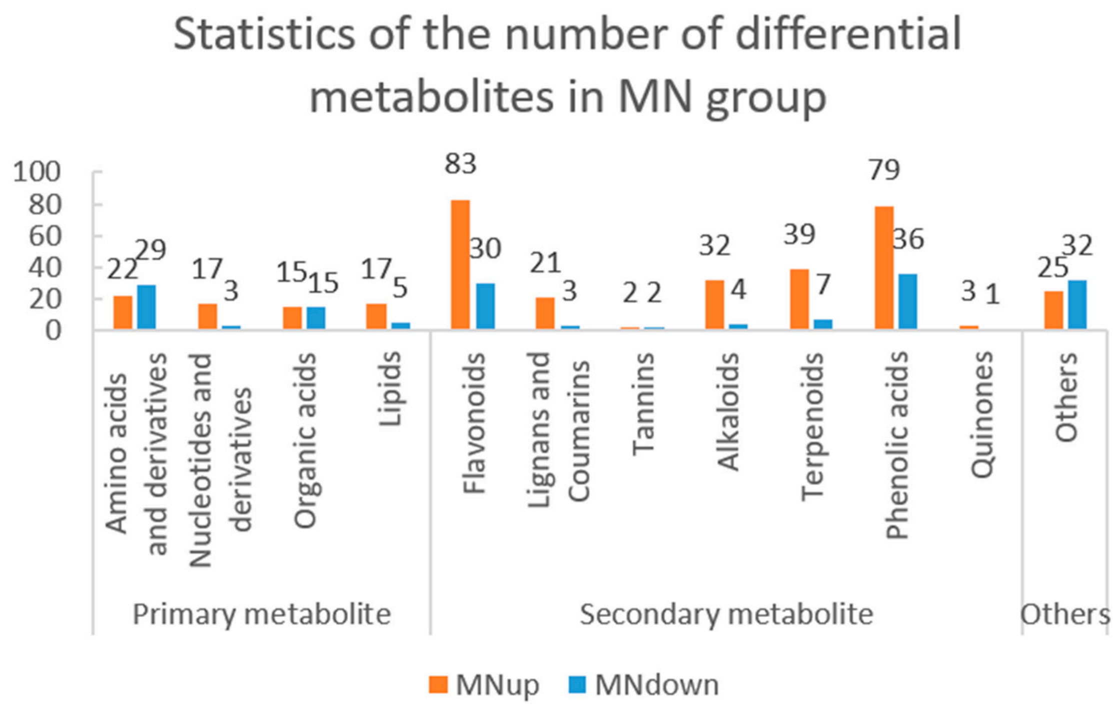

(d)

Figure S1. Differential metabolites in the MN group. (a) PCA plot of MN group;(b) volcanic map of the MN group;(c) bar graph of KEGG enrichment analysis of MN group;(d) statistics on the number of differential metabolites in the MN group. The red color in the volcano plot indicates elevated metabolite levels, green color indicates decreased metabolite levels, and the horizontal dashed line indicates a VIP value of 1, while the vertical dashed line indicates FC ≥ 1.5 or ≤ 0.67.

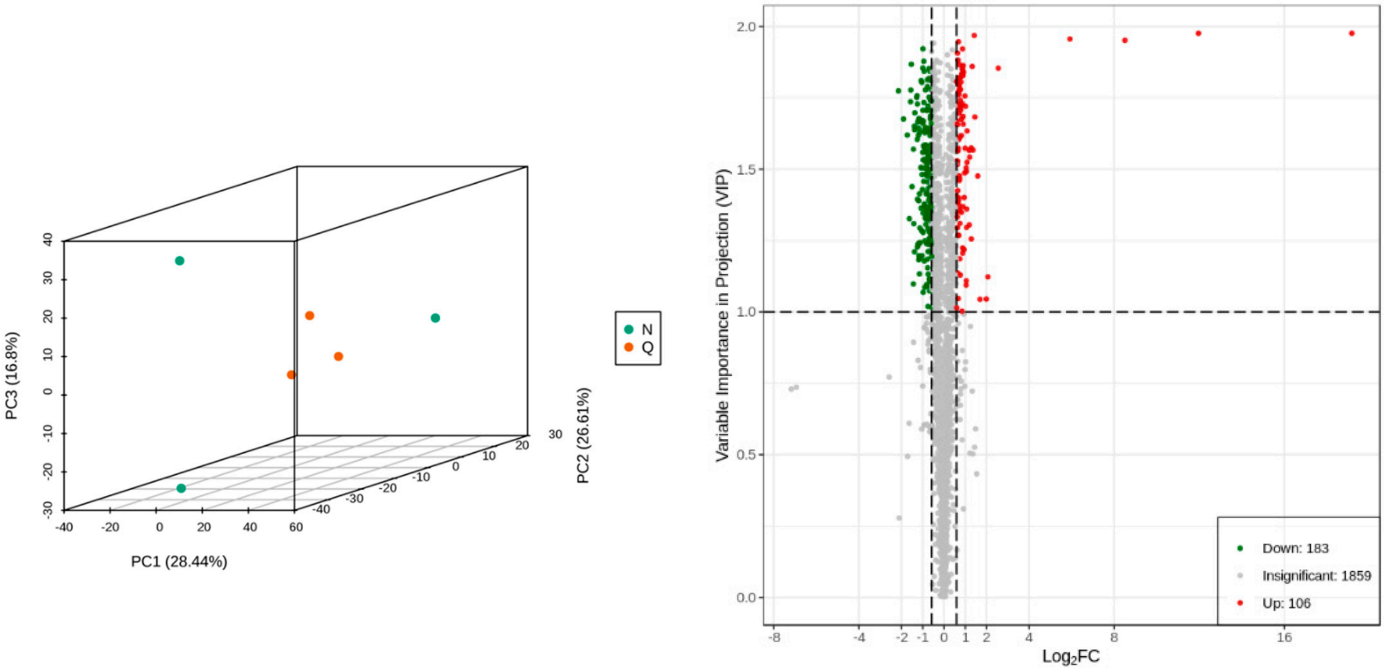

(a) (b)

Figure S2. Accumulation of phenolic acid compounds in the NQ group. (a) PCA analysis of the NQ group;(b) NQ group differential metabolite screening volcano map. The red color in the volcano plot

indicates elevated metabolite levels, green color indicates decreased metabolite levels, and the horizontal dashed line indicates a VIP value of 1, while the vertical dashed line indicates FC ≥ 1.5 or ≤ 0.67.

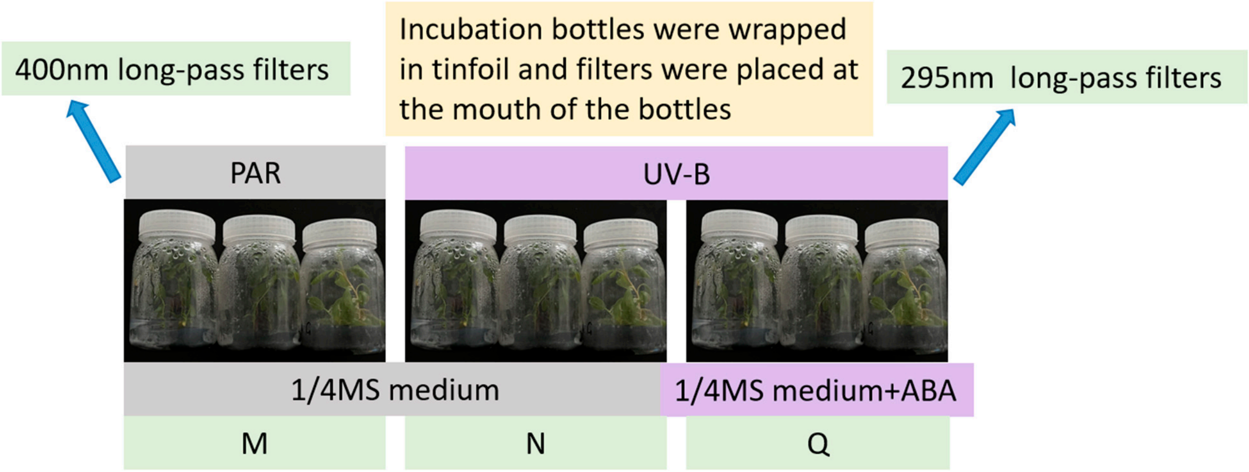

Figure S3. *R. chrysanthum* treatment. Group M was treated with PAR (400-700 nm light required by plants for photosynthesis) and group N and Q were treated with UV-B (280–315 nm). Filters with different transmittances were placed over the vials for radiation treatment, with 400 nm filters used for PAR treatment and 295 nm filters for UV-B treatment. The outside of the culture flask was wrapped in tin foil and labeled. PAR is provided by warm white fluorescent lamps and UV-B by UV-B fluorescent tubes. The radiation process lasted for 2 days (8h per day).

Table S1. Information on the names corresponding to the metabolite codes involved in the manuscript.

Index	Compounds	Index	Compounds
Ymjm000152	p-hydroxypheny-β-d-allopyranoside	pmb0751	trans-5-o-(p-coumaroyl)shikimate
Lcgn000234	davidioside C	pmn001529	3,4,5-tri-o-galloylshikimic acid
Zmtn001661	vanilloloside	Zbfn003303	3-(hydroxycinnamoyl)-quinic acid*
Lmnn102707	cimidahurinine	Lmsn000363	raspberryketone glucoside
Lmfn000797	2,3-di-o-galloyl-β-d-glucose*	Hmsn002948	2-hydroxyphenol-1-o-glucosyl(6→1)rhamnoside
pmb3068	1-o-p-coumaroylquinic acid*	Lmfn001062	2,4,6-tri-o-galloyl-d-glucose
Lmsn002887	1-o-caffeoyl-β-d-xylose	pmb3064	3-o-p-coumaroylquinic acid-o-glucoside
MWSmce248	3-hydroxycinnamic acid*	pmb3075	3-o-p-coumaroylshikimic acid
Lmbn002648	α-hydroxycinnamic acid*	Jmwn002172	3,4-dihydroxyphenylethanol-β-d-glucopyranoside

Lmfp001509	1-o-galloyl-rhamnose	HJAP046	1-(2,4,5-trimethoxyphenyl)-1,2-propanedione
Wmhn001495	1,4-di-o-galloyl-d-glucose	MWSmce712	ethyl phenylacetate
Yajn002315	4-o-(3'-o-alpha-d-glucopyranosyl)caffeoylquinic acid	Hmhn003067	phenylpropionic acid-o-β-d-glucopyranoside
Wasn005803	protocatechuic acid 4-o-(6''-o-feruloyl)glucoside	Lmhn003802	sinapoylsinapoyltartaric acid
Hmln000659	3-o-galloyl-d-glucose*	MWSmce587	2,6-dimethoxybenzoic acid
Lmhn003801	feruloylsinapoyltartaric acid	MWSslk110	helecin
Lmdn003756	methyl caffeate	Wchn003157	1,6-di-o-galloyl-d-glucose*
mws0011	syringin	Lmsn002288	1-o-caffeoyl-(6-o-glucosyl)-β-d-glucose
Zaln004057	4-caffeoylshikimic acid	Lmsn003111	1-o-p-coumaroyl-β-d-glucose
Zmln000668	monogalloyl-diglucose	Cmyp007259	poliothrysoside; nigracin
pmb3072	3-o-p-coumaroylshikimic acid-o-glucoside	Lmrn003000	2-hydroxy-3-phenylpropanoic acid
mws0027	syringic acid	Hmgn001278	verbasoside
Wafn002491	2-o-feruloylglucaric acid	Lmhn002574	caffeoyl(p-hydroxybenzoyl)tartaric acid
Lczn000058	6-o-galloyl-1-o-vanilloyl-β-d-glucose	Zmln001312	brevifolin[geranium]
pmb3056	homovanilloylquinic acid	Lajp003510	furo(2,3-f)-1,3-bewnzodioxole
MWSHC2022	glucosyringic acid	mws0467	3-(4-hydroxyphenyl)-propionic acid
Lmyp004477	evofolin B	pma3724	1-o-feruloylquinic acid
Wasn002902	o-p-coumaroylgalactaric acid	pmb2833	3-o-feruloylquinic acid-o-glucoside
Yaan002980	2,3-di-o-galloyl--d-glucose*	Hmsn002598	salirepin
pmb3074	5-o-p-coumaroylquinic acid	mad2394	sinapoyl-p-coumaroyltartaric acid
pmn001421	3-o-p-coumaroylquinic acid	Yshj000011	2,6-dimethoxy-4-hydroxyphenyl-1-o-beta-d-glucopyranoside*
MWSHC2062	ferulic β-glucoside	Zbfn004301	2-o-(4-carboxylic acid phenethyl)-6-o-caffeoyl glucoside
Lmsn003318	1-o-feruloyl-β-d-glucose	Smrp001932	(2R)-3-(3',4',5'-trimethoxyphenyl)-1,2-propanediol
HJN003	1-o-sinapoyl-β-d-glucose	Lmgn002250	dicaffeoylquinic acid-o-glucoside
Lmfn001209	1,3,6-tri-o-galloyl-β-d-glucose	MWS1830	ethylsalicylate

Jmwn006041	(7S,8R)-dehydrodiconiferylalcohol-9'-o-β-d-glucopyranoside	MWSmce454	3,5-dihydroxyacetophenone
pmn001420	1-o-caffeoyl-β-d-glucose*	MWS3136	4-methoxysalicylic acid
MWSHC20125	5-o-caffeoylshikimic acid	Lmmn002131	3'-methoxyorobol-7-o-glucoside
Lmmn001643	2-hydroxycinnamic acid*	Wmyn000213	3,4-di-hydroxyphenethyol alcohol 4-o-β-d-(6'-o-galloyol)-glucopyranoside
MWSslk066	3-hydroxy-4-methoxybenzoic acid; isovanillic acid	pmb3061	5-o-p-coumaroylquinic acid o-glucoside
Lmqn005404	4-hydroxybenzoyl-1-o-(6''-o-galloyl)glucoside	Wacn003131	2-o-p-coumaroylhydroxycitric acid
Zmhn001358	4-o-glucosyl-4-hydroxybenzoic acid	pma6460	4-o-p-coumaroylquinic acid
MWSmce675	arbutin	mws1153	2'-acetylacteoside
Jmwp003339	2-phenylethy-1-o-β-d-glucoside	pmn001537	4-o-digalloyl-3,5-di-o-galloylquinic acid
pmb0758	anisic acid-o-feruloyl glucoside	Yshj000013	4,6-dimethoxy-2-methoxyphenyl-1-o-beta-d-glucopyranoside
Zmhn000892	4-o-glucosyl-3,4-dihydroxybenzyl alcohol	Hmgp002146	leucosceptoside A
ML10179289	2-phenylethanol	Lmhn002573	sinapoylglucuronic acid
MWSprf147	glucovanillin	Zbfn002169	3'-p-coumaroyl-sucrose
Zbdn001947	isotachioside*	Hmtn001120	5-(2-hydroxyethyl)-2-o-glucosylphenol
Lmfn000604	6-o-galloyl-β-d-glucose*	MWSmce294	elemicin
pmn001320	1-o-p-cumaroylglycerol	Lmtn000940	1-o-(3,4-dihydroxy-5-methoxy-benzoyl)-glucoside
WaYn005387	populoside	Lmyn000160	mucic acid dimethyl ester
Lmln001195	picein (4-acetylphenyl-glucoside)	Lmhn003246	sinapoylcaffeoyltartaric acid
pmn001526	1,6-di-o-galloyl-β-d-glucose	Wcdp009119	elaidolinolenic acid
pma0110	4-o-sinapoylquinic acid	Wafn002827	dihydroferulic acid glucoside
Jmwn004308	4-dihydroxyphenethoxy-8-o-β-d-[6-o-(4-o-β-d-glucopyranosyl)-feruloyl]glucopyranoside	MWSslk149	5-methoxysalicylic acid
Zbfn002690	1-(4-hydroxybenzoyl)glucose; 25545-07-7	pme3443	sinapinaldehyde
Wafn002081	dihydroxybenzoyl xyloside	pmn001573	desmethylagrimonolide-6-o-glucoside*
pmb2620	3,4-dimethoxycinnamic acid	pmn001629	digalloylglucose*

mws1336	4-aminobenzoic acid	Wmyn000214	gentisic acid 5-o-β-d-(6'-o-galloyl)-gluco-pyranoside
Zmhn003082	5-o-galloyl-methyl quinine ester	Lmqp002115	methyl syringate
MWS4301	3-hydroxyphenylacetic acid	Lmdn004491	martynoside
mws1078	anthranilic acid	MWSslk083	4-hydroxy-3,5-dimethoxybenzyl alcohol
Wasn006584	1-galloyl-6-o-Benzoyl glucose	Wchn002309	3,4-di-o-galloyl-d-glucose*
mws2367	salidroside	MWS20194	cinnamic acid
Hmln002149	acteoside; verbascoside	mws0183	3,4-dihydroxybenzoic acid (protocatechuic acid)*
ML10177402	4-aminosalicylic acid	Lmyn003028	benzyl-β-gentiobioside*
Hmyn001360	doitungbiphenyl A	Lakn003294	2-β-d-glucopyranosyloxy-5-hydroxyphenylacetic acidmethylester*
Zmhn001926	1-o-salicyloyl-β-d-glucose	pmb0752	3-o-feruloylquinic acid

Table S2. Correlation analysis of abscisic acid with differential phenolic acids.

spec	env	r	spec	env	r	spec	env	r
ABA	Lmsn003318	-0.429	ABA	Lmmn001643	-0.020	ABA	Lmdn003756	0.105
ABA	mws0467	-0.404	ABA	MWSmce248	-0.005	ABA	HJN003	0.155
ABA	pma6460	-0.215	ABA	Lmbn002648	-0.005	ABA	mws1336	0.185
ABA	Lmrn003000	-0.179	ABA	pmb3074	-0.004	ABA	mws1078	0.197
ABA	pmn001420	-0.174	ABA	pmn001526	0.009	ABA	pmb0751	0.273
ABA	Lmsn003111	-0.167	ABA	ML10179289	0.032	ABA	ML10177402	0.385
ABA	MWSprf147	-0.159	ABA	mws0027	0.042	ABA	MWS4301	0.464
ABA	MWSmce675	-0.064	ABA	Hmln002149	0.059	ABA	Lmln001195	0.522
ABA	mws2367	-0.049	ABA	Lmfn001209	0.072	ABA	mws0011	0.567

Table S3. Correlation analysis of abscisic acid with 51 differential phenolic acids.

spec	env	r	spec	env	r	spec	env	r
ABA	Lmqp002115	-0.063	ABA	Wchn003157	0.206	ABA	Jmwp003339	0.502
ABA	pmn001420	-0.029	ABA	MWSmce294	0.223	ABA	Wafn002827	0.503
ABA	pmn001573	0.032	ABA	pmb3075	0.225	ABA	Lmhn003802	0.546
ABA	Lmyn003028	0.050	ABA	MWSslk083	0.253	ABA	Hmtn001120	0.578
ABA	pmb2833	0.063	ABA	Lmhn003801	0.255	ABA	ML10179289	0.589
ABA	Lajp003510	0.084	ABA	pmb3072	0.267	ABA	Yaan002980	0.600
ABA	pmb0758	0.088	ABA	ML10177402	0.273	ABA	Wchn002309	0.605
ABA	Lmdn004491	0.092	ABA	MWSprf147	0.289	ABA	Lmyn000160	0.609
ABA	MWSslk110	0.096	ABA	Lmgn002250	0.299	ABA	MWSslk149	0.637
ABA	mws0467	0.142	ABA	Lmhn003246	0.309	ABA	mws1153	0.641
ABA	Wacn003131	0.152	ABA	Lmrn003000	0.386	ABA	MWS20194	0.683
ABA	Wcdp009119	0.173	ABA	pmb0752	0.391	ABA	Lmfn001209	0.694
ABA	Lmtn000940	0.175	ABA	Lakn003294	0.398	ABA	Wmhn001495	0.706
ABA	Wmyn000214	0.179	ABA	pme3443	0.445	ABA	mad2394	0.711
ABA	Lmsn003111	0.188	ABA	Lmln001195	0.454	ABA	Lmfn001062	0.725
ABA	mws0183	0.190	ABA	pmn001629	0.465	ABA	pmn001320	0.809
ABA	Cmyp007259	0.195	ABA	MWSmce454	0.496	ABA	pmn001526	0.812

Table S4. Correlation analysis of abscisic acid with 15 differential phenolic acids.

spec	env	r	spec	env	r	spec	env	r
ABA	pmn001420	-0.029	ABA	ML10177402	0.273	ABA	Lmln001195	0.454
ABA	mws0467	0.142	ABA	MWSprf147	0.289	ABA	ML10179289	0.589
ABA	Lmsn003111	0.188	ABA	Lmrn003000	0.386	ABA	MWS20194	0.683
ABA	mws0183	0.190	ABA	pmb0752	0.391	ABA	Lmfn001209	0.694
ABA	MWSmce294	0.223	ABA	pme3443	0.445	ABA	pmn001526	0.812
